# Impact of pecking amplitude on cyclic fatigue of new nickel–titanium files

**DOI:** 10.1002/cre2.811

**Published:** 2023-12-06

**Authors:** Eugenio Pedullà, Teocrito Carlesi, Alfio Pappalardo, Francesco Saverio Canova, Vito Antonio Malagnino, Giusy Rita Maria La Rosa, Luigi Generali

**Affiliations:** ^1^ Department of General Surgery and Medical‐Surgical Specialties University of Catania Catania Italy; ^2^ Department of Medical, Oral and Biotechnological Sciences, Faculty of Medicine and Surgery University of Chieti—Pescara “G. D'Annunzio” Chieti Italy; ^3^ Department of Innovative Technologies in Medicine and Dentistry, Faculty of Medicine and Surgery University of Chieti—Pescara “G. D'Annunzio” Chieti Italy; ^4^ Private practitioner Catania Italy; ^5^ Department of Surgery, Medicine, Dentistry and Morphological Sciences with Transplant Surgery, Oncology and Regenerative Medicine Relevance University of Modena and Reggio Emilia Modena Italy

**Keywords:** dynamic cyclic fatigue, endodontics, Mtwo Minimal, pecking motion

## Abstract

**Objectives:**

The aim of this study is to determine the cyclic fatigue resistance of Mtwo Minimal in static and dynamic tests, with different amplitudes of pecking movements, at intracanal temperature.

**Materials and Methods:**

Two hundred new 25‐mm Mtwo Minimal rotary files (#10/0.035, #17.5/0.045, #25/0.05, #40/0.03, #45/0.03) were tested in static and dynamic cyclic fatigue tests at 35°C (±1°C). An artificial stainless‐steel canal was used. In the dynamic mode, axial movements were set at 1 and 3 mm. The number of cycles to fracture (NCF) was recorded and statistically analyzed by one‐way analysis of variance (*p* < 0.05).

**Results:**

The 3‐mm dynamic test showed significantly increased NCF than the other tests for the #10/0.035, #17.5/0.045, and #25/0.05 files (*p* < 0.05). The #40/0.03 and #45/0.03 files showed no significant differences in all the tests (*p* > 0.05).

**Conclusion:**

Mtwo Minimal showed higher cyclic fatigue resistance in the dynamic test than the static test, except for the larger instruments. The 3‐mm pecking amplitude increased the cyclic fatigue resistance of the smaller instruments.

## 1 INTRODUCTION

Nickel–titanium (NiTi) alloy has radically modified root canal instrumentation techniques in endodontics (Li et al., 2002) because of the greater flexibility and improved mechanical properties (Gambarini, 2001; Walia et al., 1988). However, despite their increased strength and flexibility, NiTi files are susceptible to unexpected fractures, especially when used in curved canals (Lopes et al., 2013). Fracture occurs through two primary mechanisms, often occurring simultaneously: torsion and cyclic fatigue (Plotino et al., 2012). Torsional fracture refers to the scenario in which the instrument engages the root canal walls with its tip and the shank still rotating. As a result, the torque exceeds the metal's elastic strength limit (Almeida et al., 2019; Silva et al., 2020). Cyclic fatigue results from the instrument's rotation within a curved canal, creating alternating cycles of tension and compression in the area where the bending is major (Pedullà et al., 2020; Sattapan et al., 2000; Uslu et al., 2020).

Laboratory assessments for cyclic fatigue tests can be either static or dynamic. In static tests, the instrument rotates within the canal at a constant length without any axial oscillation. In dynamic tests, the instrument goes back and forth within the canal (Lopes et al., 2010, 2013). This axial motion involves a predetermined amplitude simulating the clinical use of NiTi files, referred to as “pecking motion” (Zubizarreta‐Macho et al., 2021).

Multiple factors affect cyclic fatigue resistance including the file design and cross‐sectional area, kinematics, and amplitude of pecking motion (Dederich & Zakariasen, 1986; Gündoğar & Özyürek, 2017). Additionally, temperature is another factor that influences fatigue strength (Uslu et al., 2020). In particular, body temperature adversely affects the cyclic fatigue resistance of NiTi endodontic instruments. Thus, it is advisable to conduct cyclic fatigue investigations under conditions simulating body temperature to reproduce the intracanal environment more accurately (Savitha et al., 2022).

Several methods such as modifications in file design and operating sequence have been introduced to avoid breakage of rotating NiTi instruments and increase their metallurgical features (Gündoğar & Özyürek, 2017). The new Mtwo Minimal (Sweden Martina) is designed and marketed for use in continuous rotation with a minimally invasive sequence. They are made of a traditional NiTi alloy with the same “S italic” section of Mtwo Classic. The file sequence consists of #17.5/0.045 and #25/0.05 files, which allow preparation of the majority of root canals, according to the manufacturer (Sweden Martina, 2021). In the most complex cases, a scouting file (#10/0.035) and Mtwo Apical Minimal, available in different sizes with a reduced taper (0.03), are suggested (Sweden Martina, 2021).

To the best of our knowledge, no research is available on the cyclic fatigue of the new Mtwo Minimal instruments and, more specifically, on how different amplitudes of pecking motions can influence their cyclic fatigue resistance.

Hence, the aim of this laboratory study was to investigate the cyclic fatigue resistance of Mtwo Minimal in static and dynamic tests, considering different pecking motion amplitudes at intracanal temperature.

The null hypotheses were as follows: (i) no significant difference between static and dynamic tests was detected for each instrument, (ii) no significant difference was detected for each instrument in dynamic tests with pecking amplitudes of 1 and 3 mm, and (iii) no significant difference was detected between different files in each test.

## 2 MATERIALS AND METHODS

The laboratory study did not involve human or animal subjects. Hence, it did not require approval from the ethics committee, as previously reported (Ha et al., 2017; Klymus et al., 2019; Pedullà et al., 2018).

A total of 150 new 25‐mm‐long Mtwo Minimal rotary endodontic instruments (#10/0.035, #17.5/0.045, #25/0.05, #40/0.03, #45/.03) were used. With a sample size of 150 instruments, divided into groups of 15, a power of 0.80, and *α* = .05, the sample size was sufficient to detect a minimum effect of *f* = 0.36, considered medium to large. The software G*Power 3.1 for Macintosh (Heinrich Heine) was used for this purpose.

Each instrument was subjected to a thorough examination using a precision microscope (SZR‐10; Optika) to detect any visible signs of deformation. No instrument was eliminated. A total of 50 instruments (*n* = 10 for each size) were randomly assigned to the static group for the cyclic fatigue test at intracanal temperature. One hundred instruments were randomly assigned to the dynamic group, with 10 instruments of each size tested at two different pecking depths of 1 and 3 mm, respectively.

Cyclic fatigue tests were carried out using a customized device (La Rosa, Palermo, et al., 2021; La Rosa, Shumakova, et al., 2021; Pedullà, Canova, et al., 2022; Pedullà, Kharouf, et al., 2022), upgraded with a moving stage to perform dynamic tests. Standardized positioning of each file within the artificial canal was enabled by a fixed platform block in which the electric handpiece was placed. In addition, the platform consisted of a movable support on rails for file insertion/withdrawal that maintained the instrument perpendicular to the artificial canal during entry. A simulated stainless‐steel artificial canal 16 mm long, with an angle of 60° and a radius of curvature of 5 mm, was used.

Each file was operated with a 6:1 reduction handpiece (Sirona Dental Systems GmbH) and was tested at an intracanal temperature of 35°C ( ± 1°C). To maintain constant temperature during the experiment, a thermostat was integrated into the customized setup. A thermocouple was positioned within the artificial canal and it regulated the thermostatic resistor, ensuring that the temperature remained constant. The resistor was activated or deactivated as required to maintain the temperature within the predetermined range (La Rosa, Palermo, et al., 2021; La Rosa, Shumakova, et al.,  2021).

In the static test, the instruments were operated with continuous rotational motion at 300 rpm, and the torque was set to the maximum value. In the dynamic mode, the amplitude of axial movements was set at 1 and 3 mm, with downward and upward speeds of 100 and 200 mm/min, respectively (Myint et al., 2020). Files were rotated at 300 rpm with a torque of 1.2 Ncm. A dedicated high‐flow synthetic oil was used to lubricate the mechanical components (Super Oil; Singer Co., Ltd.).

All instruments were tested until they fractured, which was detected both visually and audibly. The time to fracture measured in seconds was recorded from the beginning of the test until the file broke. The measurements were performed by a single examiner using a digital chronometer (Picco Alba; Seiko). Video recording was also carried out to validate the previously recorded time, to avoid human errors. Cyclic fatigue resistance was expressed as the number of cycles to fracture (NCF), calculated by multiplying the rotation speed by the time in seconds (Lopes et al., 2009).

The separated instrument tip length was measured using a digital microcaliber (Digimatic; Mitutoyo Co.).

The fracture surfaces of broken files were inspected using a scanning electron microscope (SEM) (ZEISS Supra 35VP; ZEISS GmBH) to detect the topographic signs of cyclic fatigue fracture.

### 2.1 Statistical analysis

The data were normally distributed, as confirmed by the Shapiro–Wilk test. Thus, the data were statistically analyzed by one‐way analysis of variance and Bonferroni's post hoc multiple comparison test (Prism 8.0; GraphPad Software Inc.). The significance level was set at *p* < 0.05.

## 3 RESULTS

The means and standard deviations of the NCF values of cyclic fatigue resistance for each file in static and dynamic tests are shown in Table 1.

**Table 1:** Mean ± standard deviation of the number of cycles to fracture (NCF) for each instrument tested under different cyclic fatigue test conditions.

NCF			
Instruments	Static	Dynamic	
Mtwo Minimal		1 mm	3 mm
10/0.035	486^a1^ ± 64	623^b1^ ± 27	1360^c1^ ± 713
17.5/0.045	342^a2^ ± 36	439^b23^ ± 53	596^c2^ ± 75
25/0.05	355^a2^ ± 60	351^a2^ ± 72	590^b2^ ± 92
40/0.03	386^a2^ ± 47	469^a3^ ± 78	408^a3^ ± 64
45/0.03	267^a3^ ± 23	258^a4^ ± 12	323^a3^ ± 66

*Note*: The same superscript letters indicate no statistically significant differences between tests for each instrument (*p* > 0.05). The same superscript numbers indicate no statistically significant differences between the different instruments for each test (*p* > 0.05).

Dynamic tests at 3 mm showed significantly higher NCF for the #10/0.035, #17.5/0.045, and #25/0.05 instruments compared to the 1‐mm and the static tests (*p* ≤ 0.001), with no significant differences between the latter two for #10/0.035 and #25/0.05 (*p* = 1.000). The comparison between the 1‐mm and the static tests was significant for #17.5/0.045 (*p* = 0.002).

The #40/0.03 and #45/0.03 instruments showed no statistically significant difference between both the static/1 mm (*p* = 0.122 and *p* = 1.000, respectively) and the static/3 mm (*p* = 1.000) tests or between the 1‐mm and 3‐mm amplitudes for the dynamic test (*p* = 0.239 and *p* = 0.368, respectively).

In the static and 1‐mm dynamic tests, #10/0.035 and #45/0.03 showed the highest and lowest NCF, respectively (*p* < 0.001). No statistically significant difference was found between the #17.5/0.045, #25/0.05, and #40/0.03 files (*p* = 1.000, *p* = 0.462 for #17.5/045 vs. #40/0.03) in the static test. Conversely, in the 1‐mm dynamic test, #40/0.03 showed a higher NCF than #25/0.05 (*p* < 0.001), with no significant differences among #17.5/045 versus #40/0.03 (*p* = 0.471).

In the 3‐mm dynamic test, #10/0.035 had the highest NCF (*p* < 0.001), while #40/0.03 and #45/0.03 had the lowest NCF (*p* < 0.001), with no significant difference between them (*p* = 1.000). #17.5/0.045 and #25/0.05 were not statistically different (*p* = 1.000), as also #17.5/0.045 versus #45.03 (*p* = 0.681) and #25/0.05 versus #45.03 (*p* = 0.737).

**Figure 1:**
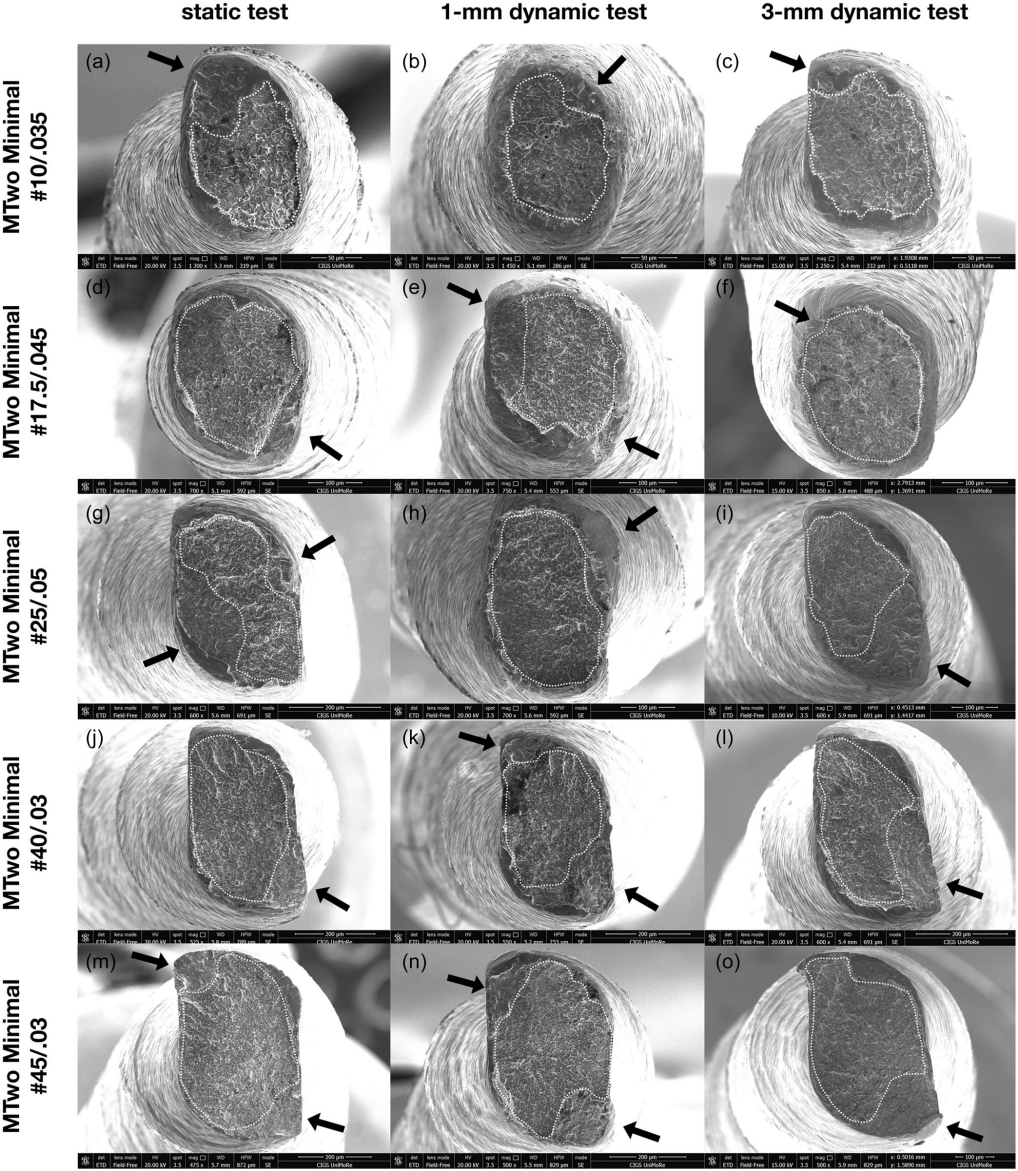
Representative field‐emission scanning electron microscopic appearances of the fractured files after static, 1‐mm, and 3‐mm dynamic tests (a–c: Mtwo Minimal #10/0.035; d–f: Mtwo Minimal #17.5/0.045; g–i: Mtwo Minimal #25/0.05; j–l: Mtwo Minimal #40/0.03; m–o: Mtwo Minimal #45/0.03). Dotted lines show the ductile fatigue area with typical microdimples and cones along the fractured cross‐sections. The origins of crack initiation are indicated by black arrows.

SEM analysis revealed comparable and typical topographic signs of cyclic fatigue fracture for all the included instruments. The crack origin and the typical surface characteristics for cyclic fatigue failure are shown in Figure 1. Furthermore, the mean length of the separated fragments did not differ significantly among the investigated files (5 ± 0.1 mm) (*p* = 1.000).

## 4 DISCUSSION

No data are currently available on the cyclic fatigue resistance of new Mtwo Minimal files due to their recent introduction (Sweden Martina, 2021). These instruments have the same traditional NiTi alloy as Mtwo Classic but have intermediate file sizes to ensure minimal and conservative root canal preparation. Thus, the purpose of this study was to assess the cyclic fatigue resistance of Mtwo Minimal files in both static and dynamic conditions with different pecking motion amplitudes at intracanal temperatures.

While natural teeth provide a more accurate representation of clinical setting, standardization is difficult due to the inherent variability of root canals (Özyürek et al., 2018). Therefore, cyclic fatigue tests were conducted in an artificial stainless‐steel canal with the canal dimensions that have been used previously (La Rosa, Palermo, et al., 2021; Pedullà et al., 2018; Pedullà, Kharouf, et al., 2022). Furthermore, the customized apparatus guaranteed standardized laboratory conditions, permitting the reproducibility of the experiments carried out (La Rosa, Palermo, et al., 2021; La Rosa, Shumakova, 2021; Pedullà, Canova, et al., 2022; Pedullà, Kharouf, et al.,  2022).

When a rotating NiTi file is used within a curved canal, the NiTi file is subjected to repetitive stresses and compressions concentrated at specific points. This repetitive stress can determine instrument fatigue and, ultimately, lead to fracture (Kitchens et al., 2007). In static tests, the file stress is concentrated in a limited file's area. These stresses cause microstructural alloy modifications (Li et al., 2002). Conversely, in dynamic tests, as the instrument moves axially along the curvature, stress is distributed across the entire file surface due to the axial movement. This increases the cyclic fatigue resistance of the file (Li et al., 2002; Thu et al., 2020).

To simulate clinical conditions and maintain the instruments in a flexed mode, pecking distances of 1 and 3 mm were selected.

According to the present results, the number of cycles required to induce fracture was significantly higher in the dynamic test than in the static test, except for the #40/0.03 and #45.03 files. Therefore, the first null hypothesis can be partially rejected. These findings might be attributed to the advantage of dynamic testing, in which stress spreads more evenly, unlike static testing, where stress is concentrated in one area of the instrument (Keleş et al., 2019; Lopes et al., 2010; Yao et al., 2006). The #40/0.03 and #45/0.03 files probably benefit less from this advantage because of their larger size, which negatively impacts their cyclic fatigue behavior. Indeed, the cyclic fatigue is negatively affected by the diameter of the instruments at the point of maximum curvature (Pedullà, Canova, et al., 2022). This mechanism occurs because all of its points (except those in the center axis) are subjected to stress/compression cycles during the instrument's rotation. The distance from the center axis is greater for larger instruments, resulting in higher stress at that point.

In addition, the differences between static and dynamic tests seem to be significantly more for the smaller‐size instruments (i.e., #10/0.035, #17.5/0.045, and #25/0.05). This could be due to the lower metal mass at the maximum stress point, which increased their cyclic fatigue resistance by allowing them to rotate for more cycles before fracturing (Pedullà, Canova, et al., 2022).

All the tested NiTi files reported greater NCF in dynamic motion with a pecking amplitude of 3 mm, except for the #40/0.03 and #45/0.03 files. Thus, the second null hypothesis can also be partially rejected. These results were obtained probably due to the fact that a larger pecking amplitude distributes the stresses over a larger area of the instrument compared with a shorter pecking depth (Li et al., 2002; Yao et al., 2006). Yet, a previous study reported that a smaller pecking amplitude generated less stress on the instrument (Ha et al., 2017). These different results are probably a result of the different methodological setup such as the type of the instruments tested. The different results obtained for #40/0.03 and #45/0.03 can be attributed again to the limitations of dynamic tests in the case of larger size instruments.

Although comparison of instruments with different tapers and sizes is challenging, some insights can be gained. Based on the current findings, the #10/0.035 instrument showed greater fatigue cyclic fatigue resistance in all tests, probably due to the advantage of small size; for similar reasons, the #45/0.03 instrument showed the lowest NCF values, corroborating the finding that files with larger diameters were more susceptible to cyclic fatigue fracture than those with smaller diameters (Pedullà, Canova, et al., 2022). The #17.5/0.045 and #25/0.05 files showed no statistically significant differences between static and dynamic tests or pecking amplitudes, likely due to a similar taper at the most stressed point. A significant difference was observed between the #25/0.05 and #40/0.03 instruments in the 1‐mm dynamic test, with the #40/0.03 instrument showing higher resistance, possibly due to the reduced taper. Indeed, increased taper at the maximum bending point has been shown to decrease the time before fracture (Pedullà, Canova, et al., 2022). Based on this, the third null hypothesis can also be partially rejected.

SEM analysis revealed that the surfaces of broken files showed comparable and typical patterns of cyclic fatigue fracture in all testing conditions, with formation of microdimples and cones along the fractured area.

The results obtained are limited to the experimental conditions tested such as the type and size of instruments as well as the pecking depth applied. Finally, several factors act simultaneously in clinical practice including the skill of the operator and anatomical variations that could modify the in vivo cyclic fatigue behavior of NiTi files.

Despite these limitations, the results are clinically significant as they confirm that a larger pecking amplitude during root canal treatment can compensate for the flexural stress on the instrument. These results reinforce the need to perform continuous pecking movements when curved root canals are fitted with rotating instruments to avoid stress concentration in one area of the instrument.

This study exhibits several novel aspects which contribute to enhance its scientific contribution. First, in this study, the cyclic fatigue of Mtwo Minimal instruments, which are relatively new and for which no prior research exists validating their mechanical properties, was investigated. Second, the utilization of a customized testing apparatus provided highly reproducible and standardized conditions, facilitating future comparative studies. Last, in this study, the influence of pecking motion amplitude on the cyclic fatigue resistance of NiTi instruments of different sizes was explored. Collectively, these novel elements significantly contribute to the existing body of knowledge and offer valuable insights for clinical practice, guiding practitioners in instrument selection and techniques for improved patient care.

## 5 CONCLUSIONS

Under the limitations of a laboratory study, the Mtwo Minimal files showed improved cyclic fatigue resistance in the dynamic test than the static test, except for larger size instruments. The 3‐mm pecking amplitude increased the cyclic fatigue resistance of smaller‐size files.

## AUTHOR CONTRIBUTIONS

Eugenio Pedullà was responsible for conceptualization, formal analysis, investigation, project administration, and writing—review and editing. Teocrito Carlesi was responsible for conceptualization, formal analysis, methodology, supervision, and writing—original draft. Alfio Pappalardo was responsible for data curation, methodology, visualization, and writing—original draft. Francesco Saverio Canova was responsible for formal analysis, validation, and writing—original draft. Vito Antonio Malagnino was responsible for conceptualization, methodology, resources, and writing—review and editing. Giusy Rita Maria La Rosa was responsible for data curation, investigation, validation, visualization, writing—original draft, and writing—review and editing. Luigi Generali was responsible for data curation, project administration, software, and writing—review and editing. All authors have contributed significantly and are in agreement with the manuscript.

## CONFLICT OF INTEREST STATEMENT

The authors declare no conflict of interest.

## Data Availability

The data that support the findings of this study are available from the corresponding author upon reasonable request.
